# How do national cultures influence lay people’s preferences toward doctors’ style of communication? A comparison of 35 focus groups from an European cross national research

**DOI:** 10.1186/s12889-015-2559-7

**Published:** 2015-12-14

**Authors:** Michela Rimondini, Maria Angela Mazzi, Myriam Deveugele, Jozien M. Bensing

**Affiliations:** Department of Public Health and Community Medicine, University of Verona, P.le LA Scuro 10-, 37100 Verona, Italy; Department of General Practice and Primary Health Care, Ghent University, De Pintelaan 185 6K3, 9000 Gent, Belgium; NIVEL (Netherlands Institute for Health Services Research), Utrecht University, Otterstraat 118-124, 3513 CR Utrecht, The Netherlands; Faculty of Social Sciences, Utrecht University, Utrecht, The Netherlands; Policlinico G.B.Rossi, UO Psicosomatica e Psicologia Clinica, P.le LA Scuro 10-, 37100 Verona, Italy; Section of Clinical Psychology, Department of Neurological,Biomedical and Movement Sciences, University of Verona, Verona, 37100 Italy

**Keywords:** National culture, Cross-cultural patient perspective, Communication skills, Doctor-patient relationship, Focus groups

## Abstract

**Background:**

The evidence that inspires and fosters communication skills, teaching programmes and clinical recommendations are often based on national studies which assume, implicitly, that patients’ preferences towards doctors’ communication style are not significantly affected by their cultural background. The cross-cultural validity of national results has been recognized as a potential limitation on how generally applicable they are in a wider context. Using 35 country-specific focus group discussions from four European countries, the aim of the present study is to test whether or not national cultures influence lay people’s preferences towards doctors’ style of communication.

**Methods:**

Lay people preferences on doctor’s communication style have been collected in Belgium, the Netherlands, the United Kingdom and Italy. Each centre organized between eight and nine focus groups, where participants (*n* = 259) were asked to comment on a video of a simulated medical interview. The discussions were audiotaped, transcribed and coded using a common framework (Guliver Coding System) that allowed for the identification of different themes.

**Results:**

The frequency distribution of the topics discussed highlights lay people’s generally positive views towards most part of doctors interventions. The regression model applied to the Guliver categories highlighted slight national differences and the existence of a cross-cultural appreciation, in particular, of five types of intervention: *Doctors attitudes* (both *Task-Oriented* and *Affective/Emotional*), *Summarizing, Structuring* and *Providing solution*.

**Conclusion:**

Lay panels valued doctors’ communication style in a similar manner in the countries selected. This highlights the existence of a common background, which in the process of internationalization of heath care, might foster the implementation of cross-national teaching programmes and clinical guidelines.

## Background

The long-lasting and multifaceted phenomenon of internationalization of heath care, has led to the development of an increasing number of educational courses that targeted at international clientele and has promoted the dissemination of cross-national medical curricula [1]. In a similar way, clinical practice has also started to implement worldwide guidelines in healthcare delivery [2]. The evidences that inspire and foster these teaching programmes and clinical recommendations, are often based on national studies. Papers are written from the implicit assumption that there are no significant differences between countries in the way doctors and patients relate to each other. However, it is unclear whether this is a valid approach. For example, a partnership doctor-communication style, that is thought appropriate in most of Western medical schools and generally advocated by Western patients in their contacts with doctors, appears to be quite difficult to apply in a South-East Asian culture. There conflict avoidance and accepted social differences leads to a one-way, paternalistic, doctors’ style of communication prevailing [3]. How far the results from such single-country studies can be transferred and how applicable these results are in other countries is hardly ever discussed as a potential limitation of the reported studies. This leads to concerns about the quality and the trans-cultural consistency of the existing evidence. Several studies have demonstrated systematic and relevant differences between countries in medical communication [4–7]. Patients’ ethnic/cultural background -meant here as the expression of belonging to a specific ethnic group - [8, 9], or their linguistic proficiency [10], have been demonstrated to have an impact on clinical outcomes and patients’ expectations. Cross-national differences in doctor-patient communication have been attributed to the characteristics of the health care system, in particular GP’s gatekeeping role [11], or to cultural difference [12]. Among the key predictors of communication factors related to culture suggested by Schouten [13], is the Hofstede model [14, 15]. It is one of the most frequently used to highlight differences in cultural values, as it enables a quantification of the dimensions that characterize this complex concept. Hofstede’s theory on national cultures, identifies four culture dimensions: power distance (PDI), uncertainty avoidance (UAI), individualism verses collectivism (IDV), masculinity verses femininity (MAS), later supplemented with a fifth dimension: long term vs short term orientation (LTO) [15].

In the present study this model will be applied in order to interpret national differences in the preferences regarding doctor’s behaviour when communicating. These were expressed by lay people in 35 country-specific focus groups, in four different European countries, Belgium, the Netherlands, United Kingdom and Italy. The immediate purpose of the study is to test whether or not cross-national differences exist. These relate both in terms of favorite topics, because they are most frequently discussed, and preferences relating to doctors’ communication performances. The ultimate aim is to provide evidence on how applicable results from single-country studies on doctor-patient communication are to other countries.

In particular the research questions explored are as follows:

1. Which are the similarities and differences between lay focus groups, by country, in the issues and topics raised during the discussions with regard to doctors’ performance in communication?
2. Which are the similarities and differences between lay focus groups, by country, in the preferences expressed regarding doctors’ performance in communication?

## Data and methods

### Participating countries

The international multicentre study draws its name (Guliver) from the four centres involved: Gent University (Belgium), Utrecht University/NIVEL (the Netherlands), Liverpool University (United Kingdom) and the University of Verona (Italy).

**Fig. 1:**
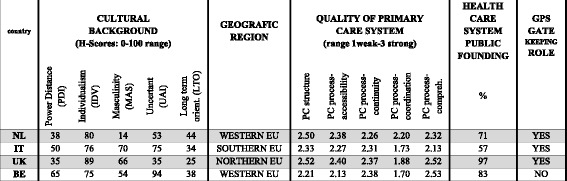
Cultural Background [15] and Health Care System [16] indicators in the four participating countries

Figure 1 shows some of the variables that describe the cultural background of the four countries from different perspectives, like *Hofstede’s dimensions* and *geographic region*.

Regarding the *geographical location* of the enrolled nations, although none of them is located in the Eastern Europe, the other three macro-regions (North, West and South) are present, assuring a wide variety of cultural backgrounds. As suggested by previous studies [11, 16, 17], *health care public funding*, *quality of primary care* and *general practitioners gatekeeping role* have been also reported in the table as possible moderator variables. These may effect modulate lay peoples’ preferences and expectations towards doctor-patient communication. In Italy and the UK, the National Health Service is based on Beveridge model [17] in which health care is provided and financed by the government through tax payments. The Netherlands and Belgium follow another model, named Bismarckmodel [17] that uses an insurance system with different health cost insurers who offer slightly different types of insurance packages.

### Panel sample

A sample of 259 participants was recruited from the general population. This was balanced by a number of factors. Firstly, age, so that at least two persons were in the classes 18–30, 31–49 and >50 years of age, for a total of 6–8 participants, in order to guarantee a heterogeneous distribution in each group. Secondly, gender, with 117 males and 142 females. Finally there was a country balance with 64 in the Netherland, 72 in Italy, 75 in UK and 48 in Belgium.

The overall sample presented a satisfactory mixture of socio-demographic characteristics: marital status (45 % married, 44 % single, other 11 %); education (13 % primary, 40 % secondary, 47 % higher school); and, occupation (57 % employed, 20 % student, 5 % unemployed, 4 % unable to work through disability, 14 % housewife/retired). The frequency distribution of these variables within each country, shows statistically significant differences in the education level (*X*^2^ 23.4 df = 6; higher school range: 36–60 % respectively for IT and UK) and occupational status (*X*^2^ 58.24 df = 12; employed: 29–87 % respectively for NL and UK). More details of the participants sample clinical characteristics are reported elsewhere [18]. Recruitment took place in public areas, via calls in free local newspaper and by word of mouth. The protocol was approved by the Medical Education Research Ethics Committee of the University of Liverpool. The written informed consent of the participants was obtained in all four countries.

### Study design and focus groups

**Fig. 2:**
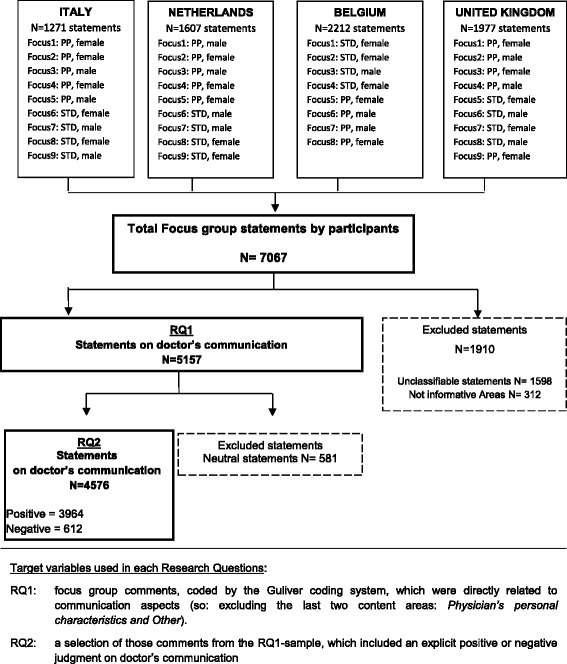
Flow chart of the study design: participants allocation to the focus groups and selected statements according to the research questions

Figure 2 illustrates the study design. A set of 35 focus group discussions (nine for each country, except Belgium with eight) were conducted following the same procedures, according to a detailed protocol [19]. Participants attended a 1-day-meeting where they watched four videotaped consultations and carried out different tasks [19]. The videotapes were standardized medical OSCE (Objective Structured Clinical Examination) consultations, in which eight different 4th year medical students from Liverpool Medical School -from now on called ‘doctors’- were assessed during their final examination. Consultations lasted on average 10 min. The maximum variation in the quality of doctors communication, was guaranteed by the combination of simulated patient ratings on a 10-point Likert scale (Global Simulated Patient Rating Scale, GSPRS) and examiners’ assessments on a checklist that included pre-established expert defined abilities defined by experts (Liverpool Communication Skills Assessment Scale, LCSAS). Two different scenarios were used, both about gynaecological problems associated with high levels of emotional distress. One was vaginal discharge related to unsafe sex- a Sexually Transmitted Disease (STD), the other was menstrual period pain (PP). As previously stated, participants were balanced by gender, anyway in order to encourage the free expression of opinions given the “gender sensitive” health problems shown in the videos, they attended gender specific focus groups.

As a prompt for the focus group discussion, participants watched four videos, based on the same scenario, in which the quality of doctors’ communication varied according to different scores of GPRS and LCSAS evaluations. Focus group discussions of 1 h followed, in which they were invited to explain their assessments from the first-round session, share their likes and dislikes regarding the doctors’ communication approach and provide underlying reasons.

### Units of analysis and measures

In order to compare the qualitative data gathered through the focus group discussions, a content analysis was performed. This aimed at creating a coding system that would allow us to synthesize, and systematically organize, participants comments. The application of quantitative techniques to qualitative data, is one of the possible use of the Mixed-Method approach [20, 21].

Each centre adopted the same set of systematic and transparent procedures for arranging and processing the raw data in order to obtain valid and reliable inferences [18]. The researchers from each centre, two from the Netherlands (J.B., L.V), three from Italy (F.M., M.R., G.D.), and one from the UK (I.F), applied an inductive content analysis of a selected set of focus group discussions. These were previously videotaped, transcribed and translated into English by researchers, who are all fluent in the English language, and checked by a native speaker. This was in order to derive a common coding framework (“Guliver coding system”) with which to classify each participant’s statement. Details about the inter-rater reliability have been published elsewhere [18].

The resulting coding system, is divided into three levels, the area, category and sub-category, to which each statement has to refer. Specific examples of the Guliver coding system categories selected in the present paper are provided in an Appendix.

When a judgment was expressed in a participants’ comment, its value was coded as positive, negative or neutral. Figure 2 indicates the variables on which the analysis have been performed in order to answer each research question.

All the focus group transcripts have been coded in their original language and also translated into English in order to make them accessible to researchers in all four centres.

### Statistical analyses

The analyses have been performed at the category level of the Guliver coding system in order to have a sufficient sample size in the comparisons between countries (Table 1 shows the consistency of the cells at the third level of classification – sub-category).

The exploration of the bivariate frequency distribution between content communication categories and participant nationality was performed using chi^2^ test and the adjusted residual analysis [22].

Two logistic regression models were estimated in order to investigate, both in terms of main effects and their interaction, the relationship between the outcome variable (positive and negative participant specific judgments) and the two independent ones which are, Guliver categories and participants’ country. Since the two independent variables are categorical, a reference category was needed for each of them; therefore C*ollecting information* was the reference category for the coding system and the Netherlands for the country. The coefficients estimated by the models were expressed according to the odds ratio interpretative approach [23]. Therefore in the first model the main effects were expressed in terms of OR (that inform on the odds of positive statements of each category in relation to a specific reference category); while the interaction effects estimated in the second model were expressed in terms of odds (that indicate the increase of positive comments for each negative produced - calculated using Margins and Marginsplot STATA commands). This methodological choice was taken in order to facilitate the interpretation of the results.

To take into account the nested structure of the study design – repeated measures within participants – the cluster option of STATA commands, was adopted in the regression models.

A count of the frequency of positive and negative statements at the level of sub-category will be provided to describe better the results obtained.

All the analysis were performed using STATA13.0 [24].

## Results

All the participants comments, stratified by judgment value (positive, neutral and negative), content (Guliver coding system – sub-category level) and country are listed in Table 1.

**Table 1:** Frequency distribution of participants’ positive, neutral and negative judgments by category and sub-category

	Country		NL			IT			UK			BE	
Category	Sub-category	Neg	Neutl	Pos	Neg	Neut	Pos	Neg	Neut	Pos	Neg	Neut	Pos
Non-verbal communication	Facial expression	2	0	6	1	5	11	0	3	3	2	1	0
	Eye contact	0	1	33	0	0	6	4	1	36	0	1	37
	Touch	0	0	1	0	0	1	0	0	1	0	0	1
	Others	7	6	15	1	7	22	25	2	17	1	5	23
	Reading and Writing	7	6	4	4	2	14	12	11	2	32	5	3
	Laughing	6	2	5	0	0	0	0	3	0	5	0	3
Structuring	Changing topics/signposting	0	1	37	4	0	7	1	0	11	5	15	17
	Flexibility	3	0	30	1	0	32	12	27	31	0	1	124
	Time issues	4	10	14	1	2	12	16	9	19	15	25	25
	Open/closing interview	0	10	31	0	2	21	11	15	26	1	8	19
Summarizing	Summarizing	1	0	44	9	0	32	10	8	25	4	2	27
Patient-involving	Sharing plans/ideas	3	0	15	1	0	18	9	15	8	5	1	20
	Asking permission	9	0	9	6	2	3	4	5	14	8	0	7
	Verifying	4	3	9	3	0	12	0	5	10	0	0	7
Speaking peculiarities	Repetition	33	1	3	9	2	1	30	3	1	9	2	0
	Fillers	14	1	4	12	0	7	11	0	0	15	0	0
	Comprehensibility	3	1	8	0	3	7	5	0	9	0	1	14
Task-oriented attitude of the doctor	Self-confident	1	0	25	1	1	47	2	15	86	0	1	52
	Complete picture	0	1	45	0	0	56	0	8	47	6	6	41
	Business-like/Straightforward	5	6	38	0	2	31	4	7	22	5	4	34
	Other attitudes	2	2	9	0	0	0	5	45	22	3	13	11
	Clarity of interview	1	0	23	0	0	18	0	5	12	0	0	19
	Competency	0	5	70	0	0	122	12	25	81	0	5	125
Collecting information	Medical	9	5	45	0	0	16	19	19	41	1	0	30
	Bio-psychological	4	8	32	1	0	19	12	9	8	4	3	24
	Psychosocial	9	7	21	6	0	24	7	13	17	31	8	28
Giving information	Medical	2	1	22	0	5	31	3	18	23	3	1	58
	Bio-psychological	3	4	17	0	1	4	2	3	18	2	0	15
	Psychosocial	1	2	0	0	1	1	2	6	10	1	0	4
Providing solution	Providing solutions	0	4	44	1	5	35	4	5	11	1	2	32
Affective emotional attitude of the doctor	Inviting attitude	1	1	25	0	0	55	0	2	34	0	0	31
	Pleasant attitude	0	0	42	0	0	32	7	9	74	0	0	43
	Show interest /commitment	0	1	36	0	0	77	1	4	44	4	4	94
	Empathetic	0	4	41	0	0	15	0	3	22	0	4	21
	Facilitating	0	0	12	0	0	36	1	0	6	0	0	54
	Reassurance / trust	2	2	89	0	1	74	1	3	36	5	2	105
	Neutral/No personal remark	12	5	38	0	2	22	3	1	2	0	5	49
	Listening	0	0	24	0	0	12	0	4	34	0	2	15
		148	100	966	61	43	923	235	311	863	168	127	1212

### Cross-national similarities and differences in the issues and topics raised during the focus group discussions

Table 2 shows the frequency distributions of focus groups comments in different countries and evidence that participants of all countries talked most about the doctors’ attitudes. Overall, about half of the comments addressed the *Task Oriented* (24 %) or, *Affective-Oriented attitudes* (27 %). The other half was focused largely on specific communication behaviours such as *Structuring* (13 %), *Collecting information* (9 %) and *Non-verbal behaviour* (8 %).

**Table 2:** Percentage frequency distribution of participant statements by country. Underlined percentage frequency showed a relevant adjusted residual (based on difference between observed and expected frequency)

Guliver coding system	Total sample		Country			
Category	Count	%	NL %	IT%	UK%	BE%
Non verbal communication	414	8.03	8.3	7.2	8.5	7.9
Structuring	653	12.7	11.5	7.8	12.6	16.9
Summarizing	164	3.2	3.7	4.2	3.5	2.2
Patient involvement	215	4.2	4.3	4.4	5.0	3.2
Speaking peculiarities	206	4.0	5.6	3.7	4.2	2.7
Task-oriented attitude	1227	23.8	19.2	26.4	28.3	21.6
Collecting information	480	9.3	11.5	6.4	10.3	8.6
Giving information	264	5.1	4.3	4.1	6.0	5.6
Providing solution	144	2.8	4.0	4.0	1.4	2.3
Affective-oriented attitude	1390	27.0	27.6	31.7	20.65	29.1
Total (count)	5157	100	1214	1027	1409	1507

National differences emerge in the comparison among countries regarding the issues and topics raised during the discussions (*X*^2^ test = 179.61, df 27; *p* < 0.01). The exploration based on the adjusted residuals allows for the identification of categories that are specifically prevalent in one of the participating countries. This is indicated by a positive gap between the observed and the expected frequencies.

Cross-national similarities emerged in the four countries during the discussion of the categories. These were: *Non-verbal behaviour* (especially the sub-category of *Other Behaviours* and *Reading and writing* -see Table 1); *Giving information* (especially the sub-category, *Medical*,); *Patient involvement,* (in particular the sub-category *Sharing plans or ideas*); and *Summarizing*.

Two topics received particular attention in the Dutch sample: *Speaking peculiarities* (6 %- in particular *Repetition*) and *Collecting information* (12 %*-* mainly due to *Biopsychological*)*.*, *Affective-oriented attitudes* (32 %)were frequently discussed among the Italian sample, in particular *Showing interest Reassuring*, *Inviting attitude* and *Facilitating*. UK citizens devoted more space to doctors’ *Task-oriented attitudes* (28 %), in particular the sub-categories *Competency* and *Self-confident.* Finally, the Belgian group talked more than the other countries about *Structuring* (17 %), especially those interventions labelled as *Flexibility* and *Time issues*.

### Cross-national similarities and differences in the participants’ preferences expressed during lay focus groups

The frequency distribution of positive and negative comments (Table 3) suggest the presence of a general appreciation of the majority of doctors interventions. The percentage of positive comments is 87 %; range: 79–88 % for UK and BE respectively.

**Table 3:** Percentage of participants’ positive judgments by Guliver Coding System-category and country

Guliver coding System	Total sample	NL	IT	UK	BE
Category					
Non-verbal behaviour	69.1	74.4	90.0	59.0	62.6
Structuring	86.0	94.1	92.3	68.5	89.8
Summarizing	84.2	97.8	78.0	71.4	87.1
Patient involvement	71.7	97.3	76.7	71.1	72.3
Speaking peculiarities	27.7	23.1	41.7	17.9	36.8
Task-oriented attitude	95.6	95.9	99.6	92.2	95.3
Collecting information	74.8	81.7	89.4	63.5	69.5
Giving information	91.4	86.7	100	87.9	92.8
Providing solution	95.3	100	97.2	73.3	97.0
Affective-oriented attitude	97.2	95.3	100	95.1	97.9
Total	86.6	86.7	93.8	78.6	87.8

The logistic regression showed that the British sample was more critical compared to Netherlands (OR = 0.46; *p* < 0.05) while Italians shown a more positive attitude (OR = 2.07; *p* < 0.05). The results for cross-cultural positive appreciation, in particular of five types of intervention were as follows: *Providing solution* (OR = 5.92 *p* < 0.05; 95 %), *Giving Information* (OR = 4.08 *p* < 0.05; 92 %),*,* doctors’ attitudes (both *Task-oriented* OR = 7.41 *p* < 0.05; 96 % and *Affective-oriented* OR = 11.37 *p* < 0.05; 97 %) and *Structuring* (OR = 2.15 *p* < 0.05; 86 %). The category that resulted less appreciated was *Speaking Peculiarities* (OR = 0.11 *p* < 0.05; 28 %).

Although, analyses were limited to the category level, due to their sample size, a description of the frequency distribution of the sub-categories (Table 1), will enable a better understanding of the categories commented on above.

Focusing on the positive comments referring to *Giving Information,* the *Medical* content was particularly valued by participants (66 %; range UK 45 % and IT 86 %), among *Task-oriented attitudes, Competency* resulted in being the most appreciated (38 %; range UK 30 % and IT 45 %). Country specific preferences emerged among *Affective-oriented attitudes*: *Reassurance was* the most appreciated by Dutch and Belgians (29 and 26 % respectively). Italians also valued interventions that *Show interest or commitment* (24 %) while British subjects regarded, positively, expressions indicating a *Pleasant attitude* of the doctor (29 %). Finally, within the category, *Structuring,* which was also valued positively by panel samples, *Flexibility* got the highest percentage of comments (48 %; range NL 27 % and BE 67 %).

On the other hand, the negative evaluation of *Speaking Peculiarities* relies mainly on the sub-category *Asking permission* (25 % of positive comments; range IT 9 % and UK 44 %).

**Fig. 3:**
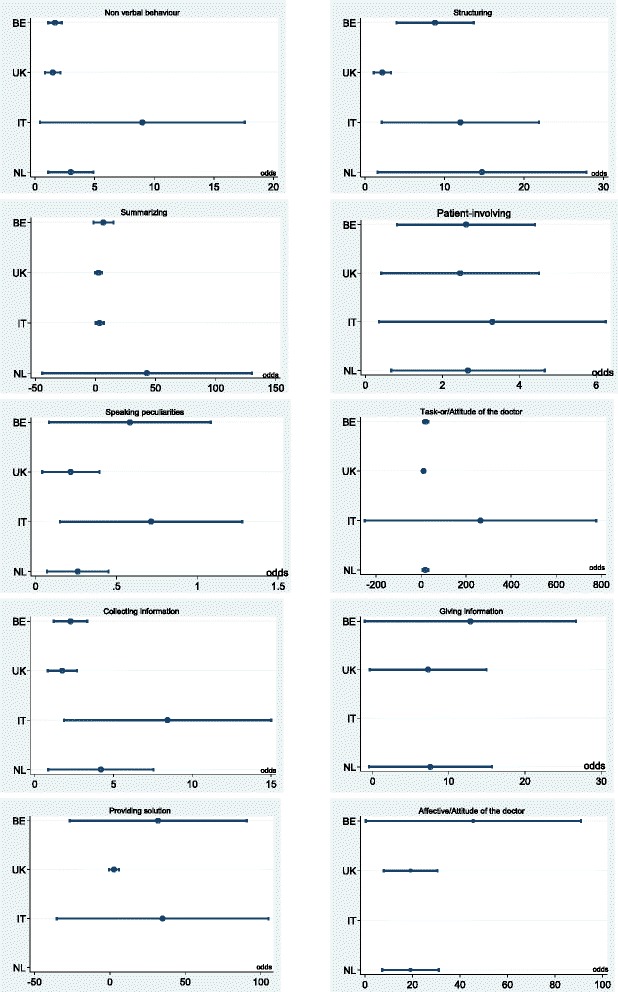
Odds and 95 % confidence interval of each Guliver category per country

Alongside the above described main effects, the second logistic regression estimated the interaction between the variables category and country, in terms of odds (see Fig. 3). The plots for each category suggest that no significant differences emerged between countries, since all the confidence intervals overlapped; the only exception is for the category *Structuring* where British sample showed a more critical attitude compared to the Belgians (Odds UK 2.2; 95%CI: 1.1;3.3 versus Odds BE 8.8; 95%CI: 3.9;13.7). In few cases, the limited number of negative comments referred to a specific category, determined not calculable or extremely wide confidence intervals.

## Discussion

The study has shown more similarities than differences in positive and critical opinions expressed by our sample of European citizens. This suggests that doctors’ performance in communication is valued more or less the same in the four participating countries. Most of doctors’ communication behaviours and attitudes were commented upon positively by the whole sample, with only few exceptions. Regression analysis indicated a cross-cultural positive appreciation in particular of four types of intervention: *Doctors attitudes* (both *Task-Oriented* and *Affective/Emotional*), *Structuring*, *Providing solution* and *Giving Information*. This combination of elements embodies a balance between doctors ability in setting up an empathetic relationship (A*ffective-oriented attitudes*) and showing competency in the solution or management of the problems and symptoms presented by the patient. Competency can be demonstrated in three ways: by interacting with the patient in a professional and self-confident manner (*Task-oriented attitudes*), by offering information and hopefully solutions to the problems presented (*Giving Information* and *Providing Solutions*) and by following a flexible approach (*Structuring*).

Hofstede’s model, and the translation of its cultural dimensions into communication styles, might offer a possible cultural explanation of this juxtaposition of communication features. All European nations here selected, scored high on the dimension ‘*Individualism vs Collectivism’*, which is characterized by a tendency towards autonomy and the exaltation of the individual and his personal resources and goals. In an earlier study, this dimension proved to be the most important in cross-national differences in doctor-patient communication [12]. This finding can be translated into a clinical approach that takes into account patient’s needs and that actively promotes his or her involvement in the decision-making process [12]. To boost this trend, there is also another cultural aspect that seems shared to a fair degree within our sample. This is the low score in the scale of ‘*Power distance’*, which denotes a validation of patient initiative and a reduction in the dominance of the doctor in the doctor-patient relationship [14]. Taking the assumption that participants moved from a common cultural background that oriented them to take an active role in the healing process, one might wonder why they choose among their favorite interventions, *Giving Information* or *Providing solution,* which can be considered directive doctor’s intervention and why *Patient involvement* did not result in being significantly more appreciated that other interventions.

The shift from the patient’s expectation of involvement and their acceptance of doctors’ suggestions may probably, have been made possible by the synergistic coexistence of the other four elements composing our five-point structure*.* Thus, patients are willing to accept solutions that come from an external source, the doctor, only to the extent that these are perceived as the fruit of a competent, flexible, empathetic and careful listening [25–27].

The wide cross-national appreciation of affective interventions does not find an immediate explanation in the scores of another Hofstede dimension usually correlated to ‘emotional expression’: *Masculinity/Femininity*. In this scale, the Netherlands is placed in the polarity *Femininity,* diverging from the other three countries with an higher grade for *Masculinity*. In communication terms, *Masculinity* is assumed to be translated in instrumental, or curing behavior, disease centred communication and biomedical talk, while *Femininity* is related to caring behavior [14]. In a previous study, using Hofstede’s dimensions for predicting cross-national differences in doctor-patient communication, the *Masculinity-Femininity* dimension also was the odd one which did not fit within the predicted pattern [12]. One possible hypothesis that might explain this absence of differences in country’s orientation towards A*ffective-oriented attitudes* based on Hofstede scores, is that cultural differences might emerge at the micro-level of specific skills used to deliver the emotional content. Indeed, participants from the Netherlands, which has the highest scores of *Femininity*, often discussed *Reassurance*, which requires a high emotional involvement of the doctor in order to be perceived as being authentic by the patient [28]. By contrast, the UK and Italy, with higher scores in the scale of *Masculinity,* were, in their comments, more oriented towards *Showing interest* and a *Pleasant attitude*, which imply a lower level of personal or emotional participation by the doctor, who is asked in this case to be polite, gentle and attentive but not necessarily compassionate [29]. However, Belgium has the same tendency to *Masculinity* as the UK and Italy have, and yet its study participants behaved more like the Dutch did, commenting positively on the way doctors practices *Reassurance.*

Of course much more can be said about the impact of national cultures on how doctor and patient communicate in the medical consultation room, and what is, or is not, appreciated by people from different countries. The literature on this issue is still scarce although the pressure to internationalize paths of care and education is growing. Our results suggest that at a macro-level, citizens present quite similar preferences towards doctors’ communication styles, and therefore, teaching programmes and clinical guidelines that stick to general recommendations might not require cultural adaptations if applied in the four countries selected here.

Anyway, previous studies [3] have highlighted that the implementation of a communicative approach (i.e.partnership relationship) in different cultures (i.e. *Western* versus *Southeast Asian*), although generally acknowledged, requires adaptations when shifting to the microlevel of doctors’ specific actions carried out during the consultation, as they can have a different impact according to the nationality of the patient. In the sample here analysed, this might be the case of doctors’ affective expressions, where how far, what patients consider, a “good intervention” can be generally applicable seems to be affected by their cultural background, and consequently, a more careful approach should be followed in the implementation of cross-national clinical and educational interventions on “patient’s emotion handling”.

International comparative cross-cultural studies based on a wider range of cultural backgrounds that assess citizens’ preferences on doctors’ communication styles, according to the specific functions of the clinical encounter, would encourage the development and promotion of culturally competent health care [30–32].

### Strength and weaknesses

The present study is based on a multi-centric dataset obtained through the fruitful collaboration of different international experts in the field of communication in medicine from Northern, Southern and Western Europe. The convenience sampling criteria limited the space to include Eastern countries in the study, which may have reduced the variety of cultural background represented. While these macro geographical divisions at the country level are, of course, too broad from an individual cultural perspective, it is yet important to analyze differences at the country level. This is because in Europe health care systems and medical curricula are organized at the country level. For policymakers as well as medical teachers it is therefore important to be aware of country-specific elements of lay people’s appreciation of doctor-patient communication.

Another possible weakness is that we were not able to check for focus group participants’ individual cultural differences, such as country of birth or religion. It is known from the literature that within countries, large cultural differences may exist between its inhabitants, which have a certain impact on doctor-patient communication [8, 9]. However, studying these individual differences was not the aim of the study.

A particular strength of this study is also that all participants had the same stimuli to react to, as they watched the identical set of videos, guaranteeing that the research for cultural differences is not contaminated by other frames of references.

The analogue patients methodology, treating laypeople as patient proxy, can be seen as a strength and a weakness at the same time. A strength is that it enables standardization of procedures and material so that the ratings are comparable (see also above). A possible limitation is that these participants are not the real patients of these doctors, which could hamper the ecological validity of the study. However, this particular methodology has been often applied in studies assessing patient perceptions [33, 34] and is recently validated [35, 36].

## Ethical approval

The project was approved by the Medical Education Research Ethics Committee of the University of Liverpool. Informed consent of the participants was obtained in all four countries.

## Conclusions

A combination of elements, emboding a balance between doctors ability in setting up an empatheticrelationship and showing competency in the management of patients’ symptoms, has been cross-culturallyvalued.Teaching programmes and clinical guidelines that stick to general recommendations, based on thiscombination of communication skills, might not require cultural adaptations if applied in the four countrieshere selected.International comparative cross-cultural studies based on a wider range of cultural backgrounds wouldencourage the development and promotion of culturally competent health care.

## Appendix

**Table 4:** Appendix: Guliver Coding System categories selected for the present paper, with examples of each subcategory

Area	Category	Subcategory	Example
Non verbal communication	Non verbal behaviour	Facial expression	*She comforted the patient with her smile*
		Eye contact	*Body language and so on, looking at the patient and so on. Someone who looks at you like this, who is turned towards you like this.*
		Touch	*..by the way he was the only one who smiled and has touched the lady before leaving. He led his hand on her arm.*
		Other behaviours	*He was fiddling with pencil like this…*
		Reading and writing	*What I find really annoying lately is the doctor sitting and typing in things on the computer while you are talking to him.*
		Laughing	*It is not really a laughable subject, so I think that I would have thought, 'Why are you laughing?*
Process-oriented expressions	Structuring	Changing topics/signposting	*She asked a lot) but sometimes I felt like, "where does it fit in?*
		Flexibility	*…he had learned his lesson by heart and he had to ask all these questions without exploring any further than that..*
		Time issues	*He says to the patient: “Time is up”. That is very impolite.*
		Open/closing interview	*I think only one of the doctors asked how the patient was like just as an informal introduction, hi how are you today, but not many of them did that.*
	Summarizing	Summarizing	*She did the summary for the patient, she wanted to be precise, to be sure of having understood.*
	Patient-involving	Sharing plans/ideas	*Asking patient what she thinks should be done now.*
		Asking permission	*Now I’d like to deepen our discussion on this topic, so if you agree I’d like to ask you few questions..*
		Verifying	*it would have been nice if, as the other three doctors have done, when patient says something the doctor in that precise moment repeats "If I understood you..”*
	Speaking peculiarities	Repetition	*He repeats his questions several times.*
		Fillers	*I did not like all these 'ok, ok,' which made me nervous.*
		Comprehensibility	*She did not complete her sentences…*
Task-oriented/problem-focused expressions	Attitude of the doctor	Self-confident	*…she was quite self-confident, I would trust her.*
		Complete picture	*I just thought he was dynamic and caught everything you know that was needed to be asked and he finished off really well.*
		Businesslike /Straight to the point	*The doctor gave straightforward answers.*
		Other attitudes	*His communication skills could have been a little better…*
		Clarity of interview	*The doctor expressed himself clearly.*
		Competency	*Competent, he has taken the time to inform you that is a reassurance. It’s a edge sort of thing, the competence of the knowledge and the way he is sharing the fact with you.*
	Collecting information	Medical	*And it was the only one who asked about a Pab test, that was good, I think. Like: did you have Pab tests before?*
		Biopsycho	*… no doctor asked whether she had had sex with her current partner recently.*
		Psychosocial	*He thoroughly explores about the job, the family is apparently less important in his opinion, or he pays less attention to it. While I think it should come first*
	Giving information	Medical	*the main thing for me is that he tells me straight away what causes the discharge.*
		Biopsycho	*she explains that she is trying to understand whether it is a psychological problem or a physical.*
		Psychosocial	*Doctors can orientate the patient and tell him: "probably the origin of the problem could be in part psychological".*
	Prov. solution	Providing solutions	*I liked her because it was interesting that she proposed how to resolve the distress of the patient at work.*
Affective/emotional expressions	Attitude of the doctor	Inviting attitude	*He has created the right atmosphere… so that the lady could say something more.*
		Pleasant attitude	*The girl had a very loving glance, a very gentle glance.*
		Show interest /commitment	*I thought that was the doctor who was most worried about the patient, who showed most involvement.*
		Empathic	*So but I liked him I put him as my favourite because I found him very empathetic.*
		Facilitating	*.…. he let her finish without interrupting…*
		Reassurance/trust	*She is my favourite. When you are the gatekeeper, then you should first of all reassure the patient that she can say anything.*
		Neutral/No personal remark	*It is not done that a GP has an opinion about it…*
		Listening	*I found him very helpful and ready to listen. He let the patient talk quite a bit..*

## Abbreviations

BE: Belgium
e.g.: *exempli gratia*
GSPRS: Global Simulated Patient Rating Scale
IDV: individualism vs. collectivism
IT: Italy
LCSAS: Liverpool Communication Skills Assessment Scale
LTO: long term vs short term orientation
MAS: masculinity vs femininity
NL: the Netherlands
OSCE: Objective Structured Clinical Examination
PDI: power distance
RQ: research question
UAI: uncertainty avoidance
UK: United Kingdom
